# 2‐month ketogenic diet preferentially alters skeletal muscle and augments cognitive function in middle aged female mice

**DOI:** 10.1111/acel.13706

**Published:** 2022-09-23

**Authors:** Suraj J. Pathak, Zeyu Zhou, Danielle Steffen, Tommy Tran, Yael Ad, Jon J. Ramsey, Jennifer M. Rutkowsky, Keith Baar

**Affiliations:** ^1^ Department of Neurobiology, Physiology and Behavior University of California Davis California USA; ^2^ Department of Molecular Biosciences, School of Veterinary Medicine University of California Davis California USA; ^3^ Department of Physiology and Membrane Biology, School of Medicine University of California Davis California USA

**Keywords:** acetylation, Alzheimer's disease, cognitive behavior, mitochondria, skeletal muscle

## Abstract

The effect of a ketogenic diet (KD) on middle aged female mice is poorly understood as most of this work have been conducted in young female mice or diseased models. We have previously shown that an isocaloric KD started at middle age in male mice results in enhanced mitochondrial mass and function after 2 months on diet and improved cognitive behavior after being on diet for 14 months when compared with their control diet (CD) fed counterparts. Here, we aimed to investigate the effect of an isocaloric 2‐month KD or CD on healthy 14‐month‐old female mice. At 16 months of age cognitive behavior tests were performed and then serum, skeletal muscle, cortex, and hippocampal tissues were collected for biochemical analysis. Two months on a KD resulted in enhanced cognitive behavior associated with anxiety, memory, and willingness to explore. The improved neurocognitive function was associated with increased PGC1α protein in the gastrocnemius (GTN) muscle and nuclear fraction. The KD resulted in a tissue specific increase in mitochondrial mass and kynurenine aminotransferase (KAT) levels in the GTN and soleus muscles, with a corresponding decrease in kynurenine and increase in kynurenic acid levels in serum. With KAT proteins being responsible for converting kynurenine into kynurenic acid, which is unable to cross the blood brain barrier and be turned into quinolinic acid—a potent neurotoxin, this study provides a potential mechanism of crosstalk between muscle and brain in mice on a KD that may contribute to improved cognitive function in middle‐aged female mice.

## Graphical abstract

Protein metabolism and inflammatory states—aging, diabetes, autoimmune disease—drive the conversion of the amino acid tryptophan to kynurenine in the liver. On a standard diet, kynurenine aminotransferase (KAT) levels are low, and kynurenine freely crosses the blood–brain‐barrier where it is converted to the neurotoxin quinolinic acid, decreasing neurocognitive function. By contrast, a ketogenic diet increases KAT levels in muscle resulting in conversion of kynurenine into kynurenic acid, which does not cross the blood–brain‐barrier, and improved neurocognitive performance.

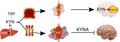

## 1 INTRODUCTION

Over the last decade, there has been increased interest in the potential therapeutic benefits of a ketogenic diet (KD). Currently, there is little known about the impact of KDs in healthy middle‐aged female mice. To date, female KD studies have primarily been completed in disease models and young mice (Nakao et al., 2019; Ruskin et al., 2017; Van der Auwera et al., 2005). In male mice, a 14‐month KD initiated in middle age (14‐month‐old) increased health span and longevity and preserved skeletal muscle mass, strength, and endurance as well as neurocognitive function (Roberts et al., 2017; Wallace et al., 2021). After 2 months on diet (16‐month‐old), male mice show the same increase in mitochondrial mass and function of a 14‐month KD but without the changes in cognitive function. By contrast, a recent study on the effect of a KD on female mice demonstrated a dramatic decrease in muscle mass and strength (Nakao et al., 2019). Whether this represents a sex difference in the response to a ketogenic diet, or simply an unpalatable, low methionine diet resulting in a starvation phenotype, remains to be determined.

The maintenance of musculoskeletal and neurocognitive function in male mice on a KD is in stark contrast to the normal decline that occurs with aging. The age‐related loss of musculoskeletal and neurocognitive function is termed sarcopenia and age‐associated cognitive decline, respectively. The decline in muscle mass and strength is a predictor of all‐cause mortality (Metter et al., 2002), whereas the incidence of neurocognitive diseases such as Alzheimer's double every 5 years between the ages of 65 and 90 (Atamna & Frey 2nd, 2007). Even with this high prevalence and the stark personal and economic cost, there are currently few treatment strategies to prevent or slow the progression of either sarcopenia‐ or age‐associated cognitive decline.

A decline of mitochondrial mass and function has been hypothesized to drive both sarcopenia‐ and age‐associated cognitive decline (Atamna & Frey 2nd, 2007; Konopka & Sreekumaran Nair, 2013). One intervention that has been shown to delay these conditions of aging is endurance exercise. Endurance exercise improves brain function through both a direct increase in brain‐derived neurotrophic factor and the activation of mTORC1 in the brain (Watson & Baar, 2014), and an indirect conversion of kynurenine to kynurenic acid (Agudelo et al., 2014) by kynurenine aminotransferases (KAT) produced in muscle. Interestingly, the signals that increase KAT activity in muscle and promote the conversion of kynurenine to kynurenic acid, are the same as those activated by exercise and a KD to increase mitochondrial mass—increased peroxisome proliferator activated receptor (PPAR) and PPARγ coactivator 1α (PGC‐1α) activity (Baar et al., 2002; Wallace et al., 2021). These data suggest that a KD may mimic exercise, increasing mitochondrial and KAT activity in muscle to improve both muscle and brain function. Since many individuals are unwilling or unable to exercise sufficiently to achieve these adaptations, interventions such as a KD that mimic endurance exercise are desperately needed to improve physical and mental function with age.

Many dietary interventions that increase longevity in model organisms are thought to mimic aspects of endurance exercise. For example, caloric restriction (Lanza et al., 2012), intermittent fasting (Weir et al., 2017), time restricted feeding (Hatori et al., 2012), and a KD (Wallace et al., 2021) increase mitochondrial mass and function. A KD, where fats are high and carbohydrates are limited, similar to fasting (Newman & Verdin, 2017) and exercise (Sleiman et al., 2016) results in the production of ketones such as β‐hydroxybutyrate (BHB) by the liver. BHB has been shown to increase lifespan in C. *elegans* (Edwards et al., 2014). In response to the rise in circulating BHB, there is an increase in acetylated protein levels—a post translational modification that can alter gene expression and protein activity, stability, or localization. A similar shift in acetylation levels is seen following exercise—another stimulus that increases fat oxidation, mitochondrial biogenesis, and neurocognitive function (Holloszy et al., 1985; Watson & Baar, 2014). Together, these data suggest that ketosis directly affects muscle and either directly or indirectly affects brain function in a way that may serve as an exercise mimetic.

Even though ketogenic diets have been proposed to improve aging, to date the majority of studies supporting this hypothesis have been performed in male mice. In contrast to the data from males, a recent study looking at the effect of a ketogenic diet on females demonstrated a dramatic decrease in muscle mass and strength (Nakao et al., 2019). Therefore, whether a KD is viable and beneficial in older females remains to be determined.

In the present study, the effect of a two‐month isocaloric ketogenic diet was compared with a control diet in middle aged female C57BL/6JN mice (starting at 14 months‐old). After 2 months on the diets, the effects on cognitive behavior, body mass, muscle mass and strength, tissue specific acetylation levels, KAT protein levels in muscle and liver, kynurenine and kynurenic acid in blood, and mitochondrial mass were determined. We hypothesized that a KD would increase acetylation levels, mitochondrial mass and KAT protein levels in a tissue specific manner resulting in a decrease in kynurenine (KYN), increase in kynurenic acid (KYNA), and enhanced cognitive performance.

## 2 METHODS

### 2.1 Animal husbandry

Female C57BL/6JN mice were obtained from the NIA Aged Rodent Colony at 12 months of age. Mice were housed in polycarbonate cages in a HEPA filtered room with controlled temperature (22–24°C) and humidity (40%–60%). Mice were maintained on a 12‐h light–dark cycle and health checks were performed daily. Health screens were completed on sentinel mice, which were housed on the same rack and exposed to bedding from the study mice, every 3 months. All tests (MHV, Sendai, PVM, MPV, MVM, M.pul and arth, TMEV [GDVII], Ectro, EDIM, MAD1 and 2, LCM, Reo‐3, MNV) were negative throughout the study. All animal protocols were approved by the UC Davis Institutional Animal Care and Use Committee and were in accordance with the NIH guidelines for the Care and Use of Laboratory Animals.

### 2.2 Experimental diets

Mice were group housed and provided ad libitum access to a chow diet (LabDiet 5001; LabDiet) until 14 months of age. Thereafter, mice were individually housed and randomly assigned to a control (CD) or a protein‐matched ketogenic (KD) diet and all mice were provided 11.2 kcal/day of CD or KD for the duration of the study. This amount was selected to match the measured chow food intake during the acclimatization period. The CD contained (% of total kcal) 10% protein, 74% carbohydrate, and 16% fat. The KD contained 10% protein, <0.5% carbohydrate, and 89.5% fat. Diets were made in‐house. The CD was a modified AIN93G diet with a lower protein content to match the KD. Table 1 provides a detailed description of the diet composition. Mineral mix TD.94046 was used for the CD, and TD.98057 was used for the KD to avoid carbohydrate carriers in the mix. We have previously demonstrated that a low carbohydrate diet containing 20% protein did not alter either skeletal muscle or neurocognitive function when compared with controls (Roberts et al., 2017).

**TABLE 1:** Composition of the experimental diets.

	Control	Ketogenic
Energy density (kcal/g)	3.8	6.7
kcal/day	11.2	11.2

Ingredients	g/kg diet	
Casein	111	191
DL‐methionine	1.5	2.7
Corn starch	490	—
Maltodextrin	132	—
Sucrose	100	—
Mineral mix TD.94046	35	—
Mineral mix TD.98057	—	24
Vitamin mix TD.40060	10	18
Soybean oil	70	70
Lard	0	582
Calcium phosphate dibasic	—	19.3
Calcium carbonate	—	8.2
Cellulose	48	85
Potassium phosphate monobasic	2.4	—
TBHQ	0.014	0.126

### 2.3 Blood ketone measurement

Blood β‐hydroxybutyrate level was measured using a Precision Xtra glucose and ketone monitoring system (Abbott) through a tail nick either 3 h postprandial or following a 12‐h overnight fast.

### 2.4 Body composition

Body composition was evaluated using NMR relaxometry (EchoMRI‐100H, EchoMRI LLC) after 2 months of diet intervention.

### 2.5 Mouse behavior tests

A series of behavior tests were performed at 16 months of age. All tests were conducted in the light cycle.

### 2.6 Barnes maze

The Barnes maze was used to assess spatial learning and memory. The maze was a white round plastic disk (92 cm diameter) with twenty holes (5 cm diameter) evenly distributed on the periphery. A black escape box equipped with a step and filled with a layer of fresh bedding was attached underneath one of the holes. The maze was raised 80 cm above the floor, and an overhead LED light source was used to illuminate the maze (700 lux). Different images were placed around the maze to be used as visual cues. In training trials, mice were placed in the middle of the maze and covered under a black bucket. The light was switched on 10 s later and the bucket was immediately lifted. Mice were allowed to explore the maze for 3 m or until they entered the escape box through the target hole. Mice were directed to the target hole and gently nudged into the escape box if they did not enter within 3 min. The light was switched off once the mouse was inside the box and the mouse was returned to the home cage after 1 min. Mice were trained with 3 trials per day for 3 days with an inter trial interval of 15–20 min. On the fourth day (probe day), the escape box was removed, and mice were allowed to explore for 2 min. Videos were recorded and analyzed using the Ethovision XT15 software (Noldus).

### 2.7 Y maze spontaneous alternation test

The Y maze was used to assess short‐term working memory. The apparatus consisted of a white acrylic Y‐shaped maze. Each arm was 35 × 8 × 15 cm (L × H × W) in dimension and was separated by a 120° angle. Mice were placed in the center of the maze and allowed to explore for 6 min. Videos were recorded, and the movement of the mice was tracked using the Ethovision XT15 software (Noldus). A complete arm entry was defined when the center point of the mouse travelled to the distal side of the arm (more than 1/3 of the arm length) and returned to the center of the maze. An alternation occurs when the mouse enters three different arms consecutively. The percent alternation was calculated as $\left(\left(\text{number of alternations}\right)÷\left(\text{number of total}\;\mathrm{arm}\;\text{entries}-2\right)\right)\times 100\%$.

### 2.8 Open field test

The open field test was conducted in a 40 × 40 × 40 cm white plastic box to evaluate mice's general locomotor activity level, anxiety, and willingness to explore. Mice were placed at the corner of the arena and videos were recorded and analyzed for 15 min using the Enthovision XT15 software (Noldus). A 25 × 25 cm middle square was defined as the center zone.

### 2.9 Novel object recognition test (NOR)

The novel object recognition test was conducted to evaluate recognition memory. This test was performed in the same apparatus as the open field test, which was also used as the acclimation session for the NOR. The familiarization session was conducted the morning following the open field test. During this session, two identical objects were placed in the arena, and mice were allowed to explore for 10 min. In the novel object session, performed 6 h after the familiarization session, one of the old objects was replaced with a novel object (the novel side was randomized among mice), and the test was performed over 10 min. A small orange cone and a cell culture flask (filled with sand) with similar height were used as the objects and were randomly assigned as the old or novel object. A mouse was considered as exploring an object if the nose of the mouse was pointed toward the object and was within 2 cm from the object. Time exploring each object was manually scored using two stopwatches.

### 2.10 Elevated plus maze

The elevated plus maze was used to assess anxiety related behavior of the mice. The apparatus consisted of a plastic plus‐shaped maze with two open arms (30 cm L × 6 cm W × 1 cm H) and closed arms (30 cm L × 6 cm W × 20 cm H) with a square center zone (6 cm × 6 cm). The maze was elevated 70 cm from the floor. Mice were placed on the center zone, facing an open arm, and were allowed to explore the maze for 5 min. Videos were recorded and analyzed with the Ethovision XT15 software (Noldus). Time on open arms was defined when the center point of the mouse traveled beyond 4.5 cm from the start of the arm.

### 2.11 Rearing test

Mice were placed into a clear acrylic cylinder (15 cm diameter) and videos were recorded for 5 min to estimate their exploratory behavior. The videos were manually scored, and a “rear” was defined as the mouse putting the forepaws on the side of the cylinder.

### 2.12 Grid wire hang test

The wire hang was used to measure skeletal muscle endurance. Briefly, mice were placed on a stainless‐steel wire mesh screen (1 mm wires and 1 × 1 cm grids) raised 40 cm above the bottom of a plastic box. Soft towels were placed at the bottom of the box to cushion the fall. Upon placing the mice on the top of the screen, the screen was slightly shaken to ensure a firm grip of the mice, and then inverted such that the mice hang on the wires using all four limbs. Time till the mice fell was recorded. If the maximal hanging time did not exceed 180 s, mice were given another trial (maximum of 3 trials) after resting in the home cage for approximately 30 min. Maximal hanging impulse was computed as (maximum hanging time (s) × body weight (kg) × 9.8 N·kg^−1^).

### 2.13 Grip strength

Mice were encouraged to grab and pull on a single metal bar attached to an Imada push‐pull force scale (PS‐500 N). Mice were given two rounds of three trials each and the maximum grip strength was used to gauge skeletal muscle strength.

### 2.14 Rotarod

Rotarod testing was used to determine motor coordination and balance. The test was conducted on a Rota Rod Rotamex (Columbus Instruments). Once the mice were placed on the rod, the rod started rotating at 4 rpm and accelerated at 1 rpm/6 s to a maximum of 40 rpm. Mice were tested for 3 trials each day for 2 days. The latency to fall and the speed at fall were recorded.

### 2.15 Euthanasia and tissue collection

Three days after the last behavior test, mice were euthanized under isoflurane anesthesia. Serum, liver, heart, hippocampus, cortex, quadriceps, soleus, gastrocnemius (GTN), and tibialis anterior muscles were collected and snap frozen in liquid nitrogen for biochemical analysis or in liquid nitrogen cooled isopentane for histological analysis. All tissues were then stored at −80°C until further processing.

### 2.16 Tissue homogenization and western blotting

Gastrocnemius, brain, and liver tissue were powdered on liquid nitrogen using a hammer and pestle. Two scoops of powder were then aliquoted into 1.5 ml Eppendorf tubes and homogenized in 250 μl of sucrose lysis buffer (1 M Tris, pH 7.5, 1 M sucrose, 1 mM EDTA, 1 mM EGTA, 1% Triton X‐100, and 1X protease inhibitor complex). The solution was set on a shaker for 60 min at 4°C, spun down at 8000 g for 10 min, supernatants were transferred to new Eppendorf tubes, and protein concentrations were determined using the DC protein assay (Bio‐Rad). Equal aliquots of 500 μg of protein were diluted in 4X Laemmli sample buffer (LSB) (final volume 200 μl) and boiled for 5 min at 100°C. 10 μl of protein sample was loaded onto a Criterion TGX Stain‐Free Precast Gel and run for 45 min at a constant voltage of 200 V. Proteins were then transferred to an Immobilon‐P PVDF membrane, after it was activated in methanol and equilibrated in transfer buffer, at a constant voltage of 100 V for 30 min. Membranes were blocked in 1% fish skin gelatin (FSG) in TBST (Tris‐buffered saline w/ 0.1% Tween) and incubated overnight at 4°C with the appropriate primary antibody diluted in TBST at 1:1000. The next day, membranes were washed with TBST for 5 min, and successively incubated at room temperature with peroxidase‐conjugated secondary antibodies in a 0.5% Nonfat Milk TBST solution at 1:10,000. Bound antibodies were detected using a chemiluminescence HRP substrate detection solution (Millipore). Band quantification was performed using BioRad Image Lab Software. Antibodies used were as followed: Total Oxidative Phosphorylation (abcam MS604‐300), PGC1alpha (ab54481), p‐AMPKthr172 (CS2531S), KAT1 (ab194296), KAT3 (sc365219), FABP1 (KAT4) (ab171739), PINK1(sc517353). LC3BI:II (cs2775S), ATG7 (cs8558S). KBHB (PTM‐1201), acetylated lysine (cs9481S), acetylated p300/CBP (cs4771), acetylated p53 (cs2570), Sirtuin 1 (cs9475S), Sirtuin 3 (cs5490S), IRE1𝛂 (cs3294), BiP (cs3183S), CHOP (cs2895S), SOD2 (sc137254), phosphorylated eif2alpha (cs3398).

### 2.17 Nuclear isolation

Cytosolic and nuclear protein fractions were extracted from frozen GTN muscles following homogenization using a glass‐on‐glass Dounce homogenizer in Buffer A (5 mM KCl, 10 mM Tris–HCL pH 7.4, 0.1% Triton X, 1.5 mM MgCl_2_, protease inhibitor complex). Samples were centrifuged at 1800×g for 5 min, and the supernatant was transferred to new tubes and labeled Cytosolic Fraction. The pellet from initial centrifugation was washed in Buffer A three times with subsequent centrifugation at 1000×g for 15 min. Following the third centrifugation, the supernatant was discarded, and the pellet was submerged in 80 μl of NET buffer (20 mM HEPES pH 7.9, 1.5 mM MgCl_2_, 0.5 mM NaCl, 0.2 mM EDTA, 20% Glycerol, 1% Triton X‐100, protease inhibitor) and sonicated for 10 s. The resuspended pellet was centrifuged at 10,000×g for 5 min and the resulting supernatent was transferred to a new tube and labeled Nuclear Fraction.

### 2.18 Enzyme‐linked immunosorbent assay (ELISA)

Kynurenine and kynurenic acid levels were measured using serum samples. The ELISAs were carried out per the manufacturers protocol (KYNA: MBS7256170, KYN: MyBioSource). KYN levels were determined after KYNA and as a result there was sufficient serum only in a subset of animals. Because of the small number of animals for the KYN measures, we fed a second cohort of mice CD or KD for 2 months and determined KYN levels to determine the reproducibility of the finding (Figure S1).

### 2.19 Total RNA and gene expression

Total RNA was isolated from frozen GTN muscle powder using Norgen Biotek Animal Tissue RNA Purification Kit (Cat # 25700) according to the manufacturer's instructions. RNA quality and quantity were measured by spectrophotometry (Epoch Microplate Spectrophotometer, BioTek Instruments). Following isolation, 1 μg RNA was reverse transcribed to cDNA using a cDNA synthesis mix (MultiScribe RT, 10x RT buffer, 10x Random Primers, dNTPs, RNase inhibitor; Applied Biosystems, Foster City, CA). Real time qPCR (CFX384 Touch Real‐Time PCR Detection System; BidRad) was performed with each sample amplified in triplicate using SYBR green as the fluorescent reporter (BioRad; PCRbio.com, Wayne, Pennsylvania). Expression of Ppargc1a, Sirt1, Tfam, and Mfn1 (Invitrogen, ThermoFisher) was determined using 2^−ΔΔCt^ method with B2m (IDT) as housekeeping control. The results are presented relative to average ΔCt of the control group.

### 2.20 Statistical analysis

All values are expressed as mean ± SEM p values <0.05 considered significant (*p < 0.05, **p < 0.01, ***p < 0.001). All analyses were performed using GraphPad Prism 8.1 (GraphPad Software Inc.) For behavior tests, comparisons between the CD and KD group were conducted using an un‐paired t‐test or a non‐parametric Mann–Whitney test. Outliers were excluded following a ROUT test. Gene expression data (2^−ΔΔCt^) was normally distributed with no outliers and analyzed with an un‐paired t test with Welch's correction.

## 3 RESULTS

### 3.1 Isocaloric feeding of a KD produced higher β‐hydroxybutyrate levels without alterations in body weight in middle‐aged female mice

**FIGURE 1:**
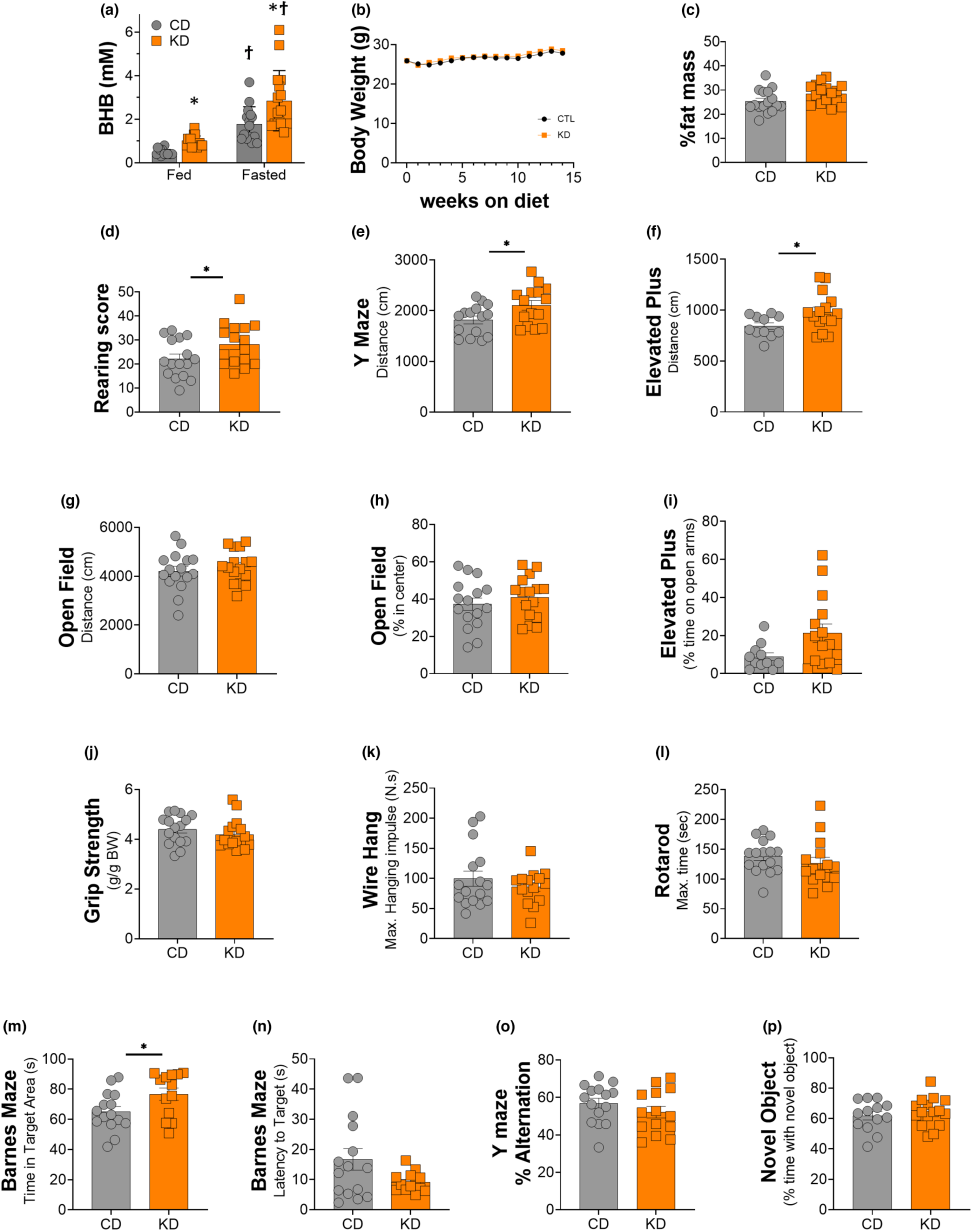
Blood ketone levels, body weight, heart, liver, and muscle mass after 2 months of a KD. (a) 3‐h postprandial and 12‐h fasted blood β‐hydroxybutyrate levels in middle‐aged female fed a control (CD) or ketogenic diet (KD). (b) Body weight, (c) percent fat measured using NMR relaxometry, (d) rearing score: Number of wall‐contact rears. Total distance travelled in (e) Y maze, (f) elevated plus maze, and (g) open field. Percent time spent (h) in center region of the open field and (i) on the open arms of the elevated plus maze. (j) Grip strength test: Relative to body weight. (k) Grid wire hang: Maximum hanging impulse. (l) Rotarod: Maximum time on the rod. (m) Time spent in the target quadrant and (n) latency to find the target hole in the banes maze probe trial. (o) Percent alternation in the Y maze spontaneous alternation test. (p) Percent time exploring the novel object in the novel object recognition time. Of female mice fed an isocaloric CD or KD. *indicates *p* < 0.05, **p < 0.01. All values are presented as mean ± SEM (*n* = 16).

Female mice on a KD exhibited significantly elevated levels of postprandial and fasting blood BHB levels compared with mice fed the CD (Figure 1a). Both the CD and KD mice gained weight over the course of the study (CD: +1.93 g, KD: +2.54 g), but there was no significant difference in body weight between diet groups at any time point (Figure 1b). Interestingly, there was a trend toward increased percent body fat mass in KD mice (*p* = 0.06, Figure 1c) compared with CD mice, and this might contribute to the slightly higher (not significant) body weight observed in the KD group. Mass of the gastrocnemius (GTN), quadricep (QUAD), and soleus (SOL) muscles showed no differences between the two groups, while the tibialis anterior (TA) tended to be higher in the KD fed mice (*p* = 0.065) (Table 2). Lastly, there was a small but significant decrease in liver weight amongst KD fed animals (Table 2).

**TABLE 2:** Tissue weights after 2 months on a control diet or ketogenic diet

Tissue	Control diet (g)	Ketogenic diet (g)
Liver	1.093 ± 0.0469*	0.9432 ± 0.08265
Heart	0.1458 ± 0.0059	0.1434 ± 0.0042
Quadricep	0.16 ± 0.0046	0.1622 ± 0.0045
Tibialis Anterior	0.03611 ± 0.0025	0.04151 ± 0.0010**
Gastrocnemius	0.127 ± 0.0019	0.1238 ± 0.0015
Soleus	0.0079 ± 0.0003	0.0079 ± 0.0003

*Note*: Values are presented as mean ± SEM.

* *p* < 0.05.

** *p* < 0.1.

### 3.2 A 2‐month KD increased exploratory behavior and some measures of locomotor activity in healthy middle‐aged female mice

Female KD mice were more active in the rearing tests (Figure 1d), consistent with an increased locomotor activity and willingness to explore. The total distance travelled on the Y maze and elevated plus maze was also significantly greater in female mice fed a KD (Figure 1e,f), although no difference was observed in the open field test (Figure 1g).

Although the percent time spent in the center region of the open field, a measure of anxiety, was not altered with a 2‐month KD (Figure 1h), there was a trend for increased time on the open arms of the elevated plus maze (*p* = 0.07, Figure 1i), consistent with reduced anxiety on the elevated plus maze or increased willingness to explore the open arms.

The 2‐month KD did not impact performance in grip strength (Figure 1j), grid wire hang (Figure 1k), or rotarod (Figure 1l). These results suggested that neither motor coordination nor strength/endurance were altered in healthy middle‐aged female mice fed a short‐term KD.

### 3.3 Spatial learning and memory were improved in female mice after 2 months on a KD, but no changes in recognition memory or short‐term working memory were observed

A significant increase in time spent in the target quadrant (Figure 1m) and a strong trend toward a decreased latency to find the target hole (*p* = 0.06, Figure 1n) in the Barnes maze probe trial were observed in female KD mice, suggesting improved spatial learning. Neither short‐term working memory measured through the Y‐maze spontaneous test (Figure 1o) nor recognition memory evaluated using the novel object recognition test (Figure 1p) was impacted by 2 months of a KD.

### 3.4 Increased acetylation within the liver is not associated with mitochondrial biogenesis

**FIGURE 2:**
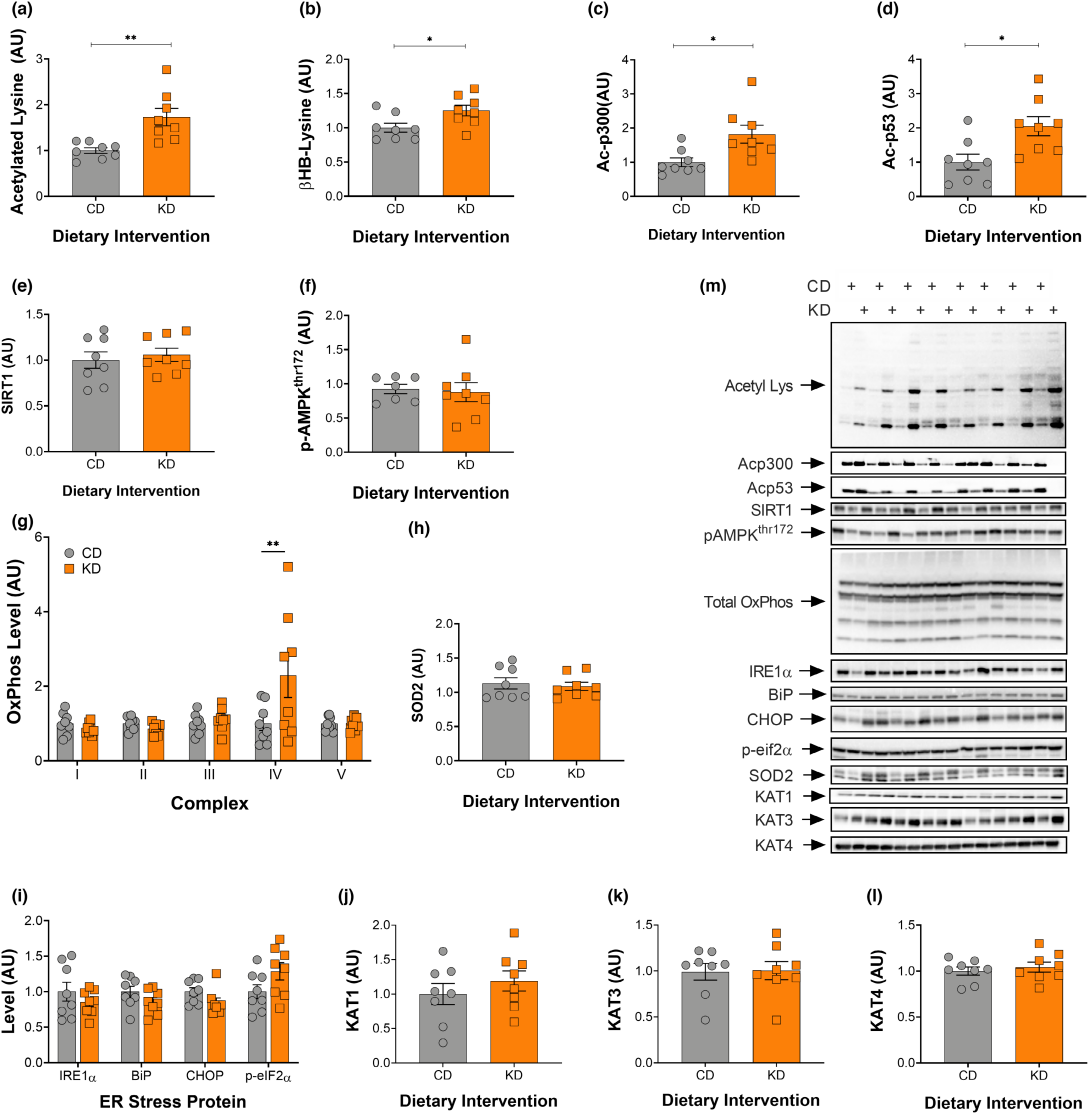
Increased acetylation in liver is not associated with mitochondrial biogenesis after 2‐months on a ketogenic diet. Quantification of (a) acetylated lysine, (b) BHB‐lysine, (c) acetylated p300, (d) acetylated p53, (e) SIRT1, (f) phosphorylated AMPK^thr172^, (g) total oxidative phosphorylation, (h) SOD2, (i) IRE1, BiP, CHOP, and phosphorylated eIF2α, (j) KAT1, (k) KAT3, and (l) KAT4 levels. (m) Representative images for all Western blot data are shown. CD, control and KD, ketogenic diet animals. *p < 0.05, **p < 0.01. All values are presented as mean ± SEM (*n* = 8).

Acetylated and β‐hydroxybutyrylated lysine (βHB‐Lysine) moieties were measured via Western blotting and found to be significantly increased, by 70% and 25%, respectively, in female KD mice when compared with controls (Figure 2a,b). Acetylation of the acetyl transferase p300/CBP was 2‐fold higher (Figure 2c) with its direct target p53 showing similar increases (Figure 2d) in the KD mice. There was no change in SIRT1 protein in the liver as a result of the diet (Figure 2e). The phosphorylation of AMPK at Thr172 was also unchanged (Figure 2f). As an estimate of mitochondrial mass, proteins in the oxidative phosphorylation pathway were measured. Only proteins from complex IV were increased in the liver of female mice on a KD (Figure 2g). There were no significant changes in markers of the integrated stress response, IRE1α, BIP, CHOP, p‐eIF2α, and SOD2 (Figure 2i,h) between diet groups, indicating that the diet was well tolerated in female mice, irrespective of the decreased liver weight. There was no difference in kynurenine aminotransferase levels (Figure 2j–l).

### 3.5 Increased acetylation within the gastrocnemius is associated with mitochondrial biogenesis and increased KAT levels

**FIGURE 3:**
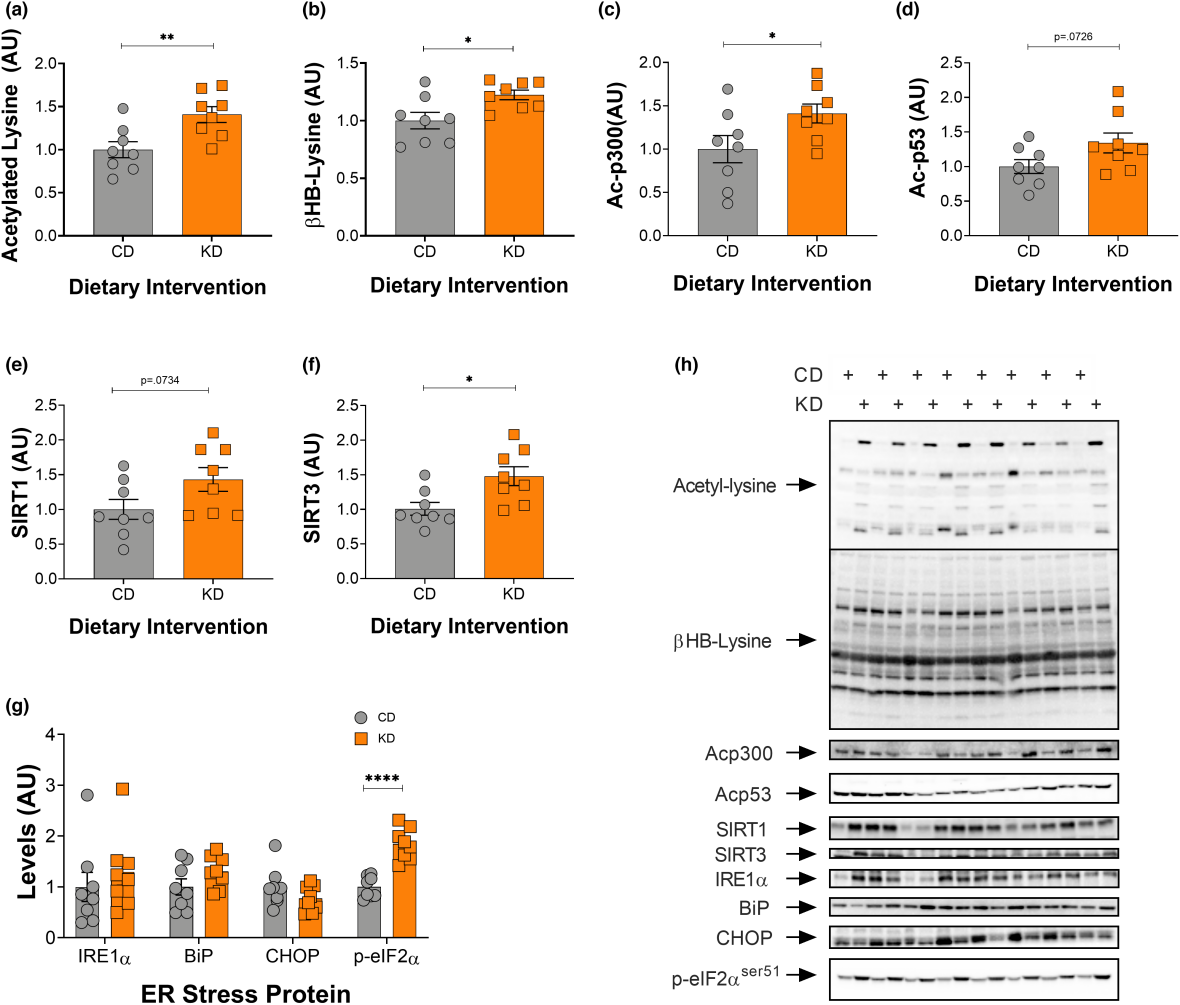
Acetylation and BHBylation increase in response to a 2‐month KD in gastrocnemius muscle. (a) Acetylated lysine, (b) BHB‐lysine, (c) acetylated p300, (d) acetylated p53, (e) SIRT1, (f) SIRT3, and (g) IRE1, BiP, CHOP, and phosphorylated eIF2α levels in the GTN muscle following 2 months on a KD. (h) Representative images for all graphs. CD, control and KD, ketogenic diet animals. *indicates *p* < 0.05, **p < 0.01, ***p < 0.001, ****p < 0.0001. All values are presented as mean  SEM (*n* = 8).

To understand how different sets of highly metabolically active tissues are impacted by the ketogenic diet—acetylation levels and mitochondrial mass was also determined in the gastrocnemius (GTN) muscle. Similar to the liver, acetylated lysine, βHB‐lysine, and ac‐p300/CBP increased in female KD mice compared with their control counterparts (Figure 3a–c). Ac‐p53 protein levels also tended to increase (*p* = 0.0726) on a KD (Figure 3e). SIRT1 showed a strong trend to increase (*p* = 0.0734), whereas SIRT3 increased significantly (Figure 3d,f) in the KD mice. ER stress markers were unchanged in the GTN (Figure 3g) with the exception of phosphorylation of eIF2α which increased 2‐fold in female KD mice (Figure 3g).

**FIGURE 4:**
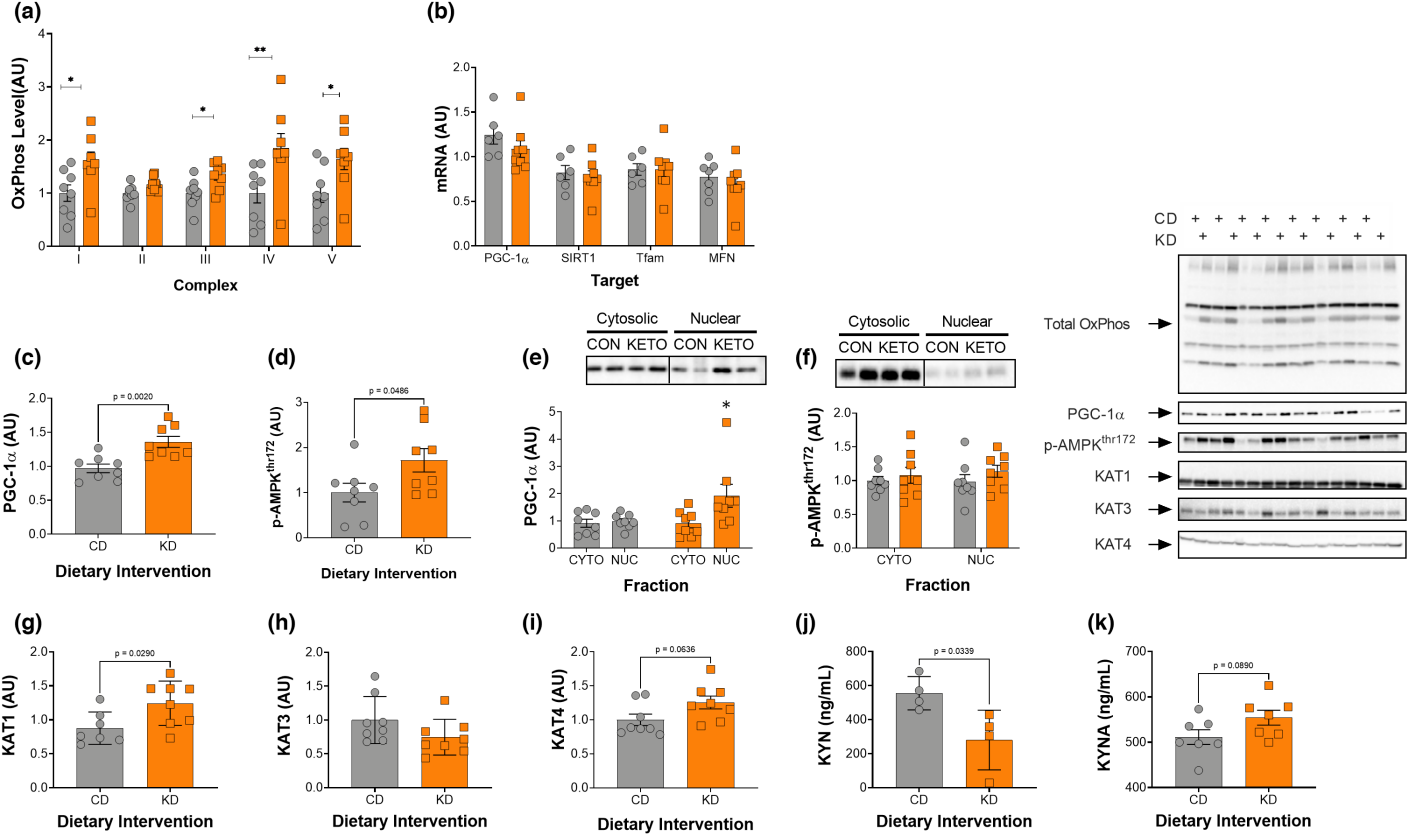
A 2‐month KD increases mitochondrial proteins, PGC‐1α, phosphorylation of AMPK, and KAT1 in gastrocnemius muscle. (a) Total OxPhos protein, (b) PGC‐1α, SIRT1, TFAM, and mitofusin expression, (c) PGC‐1α protein, (d) phosphorylated AMPK^thr172^, (e) nuclear PGC‐1α and (f) AMPK, (g) KAT1, (h) KAT3, (i) KAT4, (j) circulating kynurenine (KYN), and (k) kynurenic acid (KYNA) levels, and (k) representative images for all Western blot data is shown. CD, control and KD, ketogenic diet animals. *p < 0.05, **p < 0.01. All values are presented as mean ± SEM (*n* = 8).

Total OxPhos levels were quantified in the GTN muscle. Proteins within complexes I/III/IV/V increased significantly in the female KD mice, whereas complex II showed a strong tendency (*p* = 0.0625) to increase (Figure 4a). In the GTN, both total PGC‐1α and p‐AMPK^thr172^ were significant higher in the KD fed animals (Figure 4c,d). Additionally, female KD mice showed significantly greater nuclear localization of PGC‐1α (Figure 4e). KAT1 and KAT4 increased on a KD, whereas KAT3 remained unchanged (Figure 4g–i). Concomitant with the increase in KAT protein in muscle, plasma KYN levels decreased ~50% (*p* = 0.03) in KD relative to mice on a CD (Figure 4j and Figure S1) whereas kynurenic acid, though not significant, showed a strong trend to increase on a KD (Figure 4k). Since PGC‐1α has been implicated in controlling mitochondrial autophagy (mitophagy), PINK1, LC3B II:I, and ATG7 proteins were quantified and there were no differences between the diet groups (data not shown).

### 3.6 Selected mitochondrial gene expression is unchanged in skeletal muscle

Despite an increase of total and nuclear PGC‐1α protein (Figure 4c,e), there was no difference in the expression of *Ppargc1a* in the GTN between the control and ketogenic diet groups (Figure 4b). This may suggest that post‐translational modifications underlie the increase in total and nuclear PGC‐1α. As with PGC‐1α, there was no difference in expression of *Sirt1* or genes downstream of PGC‐1α (*Tfam* and *Mfn1*) between the diet groups (Figure 4b).

### 3.7 KAT4 specifically increases in response to a KD in soleus muscle

**FIGURE 5:**
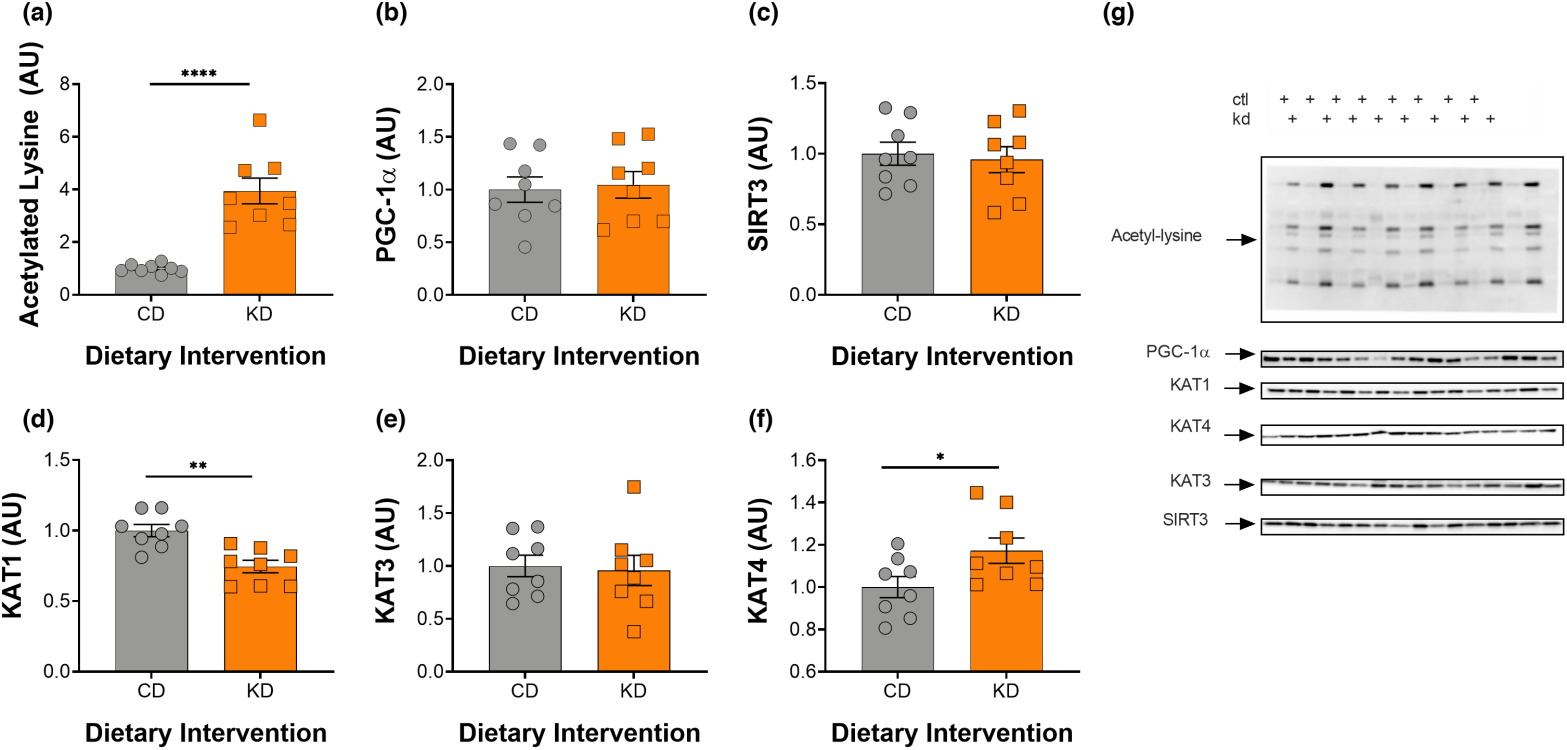
A 2‐month KD increases acetylation and KAT4 levels in soleus muscle. (a) Acetylated lysine, (b) PGC‐1α, (c) SIRT3, (d) KAT1, (e) KAT3, (f) KAT4 levels, and (g) representative im. CD = control and KD = ketogenic diet. *p < 0.05, **p < 0.01, ****p < 0.0001. All values are presented as mean ± SEM (*n* = 8).

To understand how a KD affects skeletal muscle with differing fiber type composition, the soleus muscles from the mice were prepared for Western blot analysis. As expected, levels of acetylated lysine increased significantly in the KD fed mice when compared with controls (Figure 5a). There was no increase in PGC‐1α (Figure 5b) or SIRT 3 (Figure 5c) in the more oxidative soleus muscle. Unlike the GTN muscle, KAT4 was the only KAT protein to significantly increase in the soleus while KAT1 significantly decreased (Figure 5d–f), suggesting muscle‐specific adaptations of different KAT isoforms.

### 3.8 Changes in cognitive behavior occur irrespective of changes in brain acetylation and mitochondrial OxPhos protein levels

**FIGURE 6:**
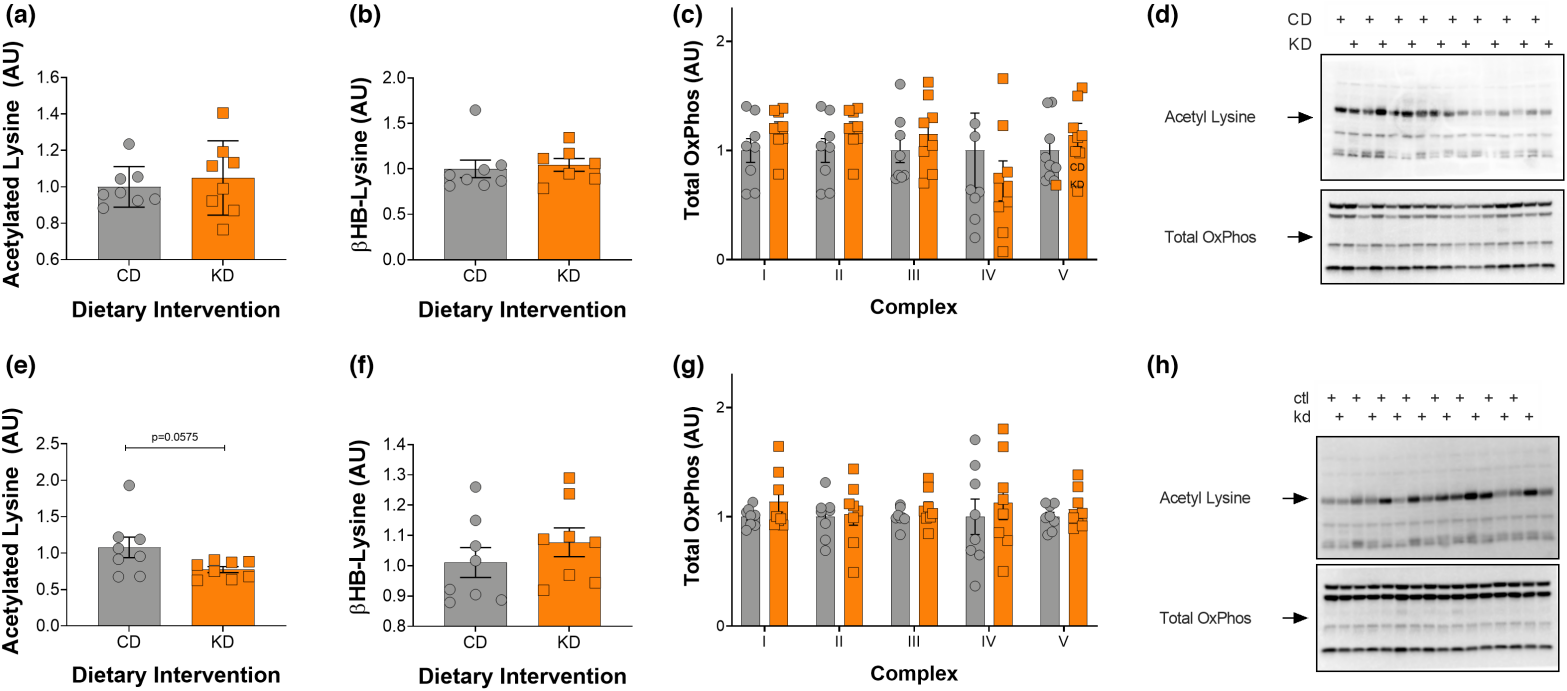
Improvements in cognitive behavior are independent of acetylation or mitochondrial levels in the cortex and hippocampus. (a) Acetylated lysine, (b) BHB‐lysine, (c) Total Oxphos, and (d) representative Western blots from the cortex. (e) Acetylated lysine, (f) BHB‐lysine, (g) Total Oxphos, and (h) representative Western blots from the hippocampus. CD, control and KD, ketogenic diet animals. All values are presented as mean ± SEM (*n* = 8).

In the cortex, there was no change in acetylated proteins between female control and KD mice, whereas in hippocampal tissue there was a strong trend toward a decrease in protein acetylation (*p* = 0.0575) (Figure 6a,e). Further, no changes were seen in BHB‐lysine levels between the groups (Figure 6b,f). Lastly, representative OxPhos proteins in both the cortex and hippocampus were unchanged by a 2‐month KD (Figure 6c,g).

## 4 DISCUSSION

The objective of this study was to determine whether a ketogenic diet (KD) negatively affected muscle mass and function in female mice. A secondary goal was to determine the neurocognitive effect of a short‐term ketogenic diet in middle aged mice. Our data on 14‐month‐old female mice that were on 2 months of a 11.2 kcal/day control or KD, shows that a KD is well tolerated in female mice and preferentially alters skeletal muscle metabolism leading to increased acetylation of lysine residues, mitochondrial mass, and kynurenine aminotransferase (KAT) levels. Further, these changes in muscle function occurred in concert with a concomitant increase in kynurenic acid in the blood and improvement in neurocognitive behavior related to memory and anxiety.

Thus far, studies on KD fed female mice have produced equivocal results regarding body weight maintenance. Previous reports include an increase (Holcomb et al., 2021; Ruskin et al., 2017), decrease (Nakao et al., 2019; Van der Auwera et al., 2005), or unchanged (Wu et al., 2019) body weight. Nakao and colleagues had recently reported that a KD can induce severe weight loss and skeletal muscle atrophy in young female mice (Nakao et al., 2019). Here we show that middle‐aged female mice on an isocaloric KD maintain body weight, muscle mass, and muscle function much the same as male mice (Fujikura et al., 2021; Roberts et al., 2017; Zhou et al., 2021). It is important to note that the KD used by Nakao and colleagues was low in protein and may have been deficient in methionine, which has been shown to be a primary cause of body weight and lean mass loss observed on low‐protein KDs (Pissios et al., 2013).

Treatments that shift metabolism, such as caloric restriction, exercise, and a KD, enhance mitochondrial mass and activity and extend life span (Mercken et al., 2012; Roberts et al., 2017; Weindruch et al., 2001). Common amongst these three interventions is an increase in acetylation—an important post translational modification. The removal of the positive charges within histones decreases their interaction with negatively charged DNA, resulting in more open chromatin and increased transcriptional activity (Reinke & Hörz, 2003). In non‐histone proteins, the charge change can result in a change in shape that alters the location, activity, or stability of the protein (Christensen et al., 2019). We have previously demonstrated that in response to a KD there is an ~6‐fold increase in acetylated lysine levels in liver tissue in male mice (Roberts et al., 2017). Here, we measured a smaller ~2‐fold increase in acetylated lysine in the livers of female mice following 2 months on a KD (Figure 2a). There were no signs of mitochondrial biogenesis in the liver in response to the increase in acetylation, except for a small but significant rise in complex IV (Figure 2g). This is consistent with recent findings published by our group, showing that male mice on a KD for 1‐month had no difference in liver mitochondrial complex I activities, whereas a significant change in complex IV activity was observed (Zhou et al., 2021).

In line with our hypothesis, a KD increased acetylation levels, mitochondrial mass, and KAT protein levels in the GTN muscle of middle‐aged female mice. The effects of a KD on acetylation and mitochondrial mass and activity mirrors those of middle‐aged male mice on a KD (Roberts et al., 2017; Wallace et al., 2021) or those treated with the HDAC inhibitor scriptaid, including increased fat oxidation and mitochondria in skeletal muscle (Gaur et al., 2016). These results suggest that acetylation may be an important physiological outcome of a KD. In support of this hypothesis, the increased mitochondrial mass on a KD was associated with greater acetylation of histone acetyltransferase protein (HAT) p300 and its direct target p53 within the gastrocnemius (GTN) muscle (Lill et al., 1997). Acetylation of p300 on the 17 lysine residues within its regulatory domain stimulates acetyltransferase activity and enhances the ability to interact with target proteins (Karanam et al., 2007; Karukurichi et al., 2010). The importance of p300 in skeletal muscle was recently demonstrated in mice with a skeletal muscle‐specific knockout of p300 who showed decreased grip strength and rotarod performance without histological evidence of disease (Svensson et al., 2020). Together, these data suggest that p300 may be a candidate for future studies regarding the KD and muscle function with age. One way that a KD could mediate the effects of p300 in skeletal muscle is through opening chromatin around myogenic regulatory factor protein (MyoD) binding sites (Tapscott, 2005). Since these sites are crucial for skeletal muscle development, function, and regeneration, KD‐dependent increases in p300 activity could underlie some of the benefits of the diet on muscle mass and function with age. It is important to note that no differences were seen in grip strength, wire hang, or rotarod performance between the diet groups. This finding is similar to what we see with 2 months of a KD in middle aged male mice (Roberts et al., 2017). By contrast, grip strength and wire hang improved in 26‐month‐old male mice who had been on a KD for 14 months, suggesting that the functional benefits of the diet may be more pronounced in older animals where loss of strength and endurance is normally observed. The acetylation of p53, which is fundamental for its activity, complex assembly and therefore its cellular effects (Tang et al., 2008), was increased in the GTN muscle of female mice, as previously demonstrated in male mice (Roberts et al., 2017). Active p53 can move to mitochondria where it stabilizes mtDNA and prevents mtDNA damage (Liang & Clarke, 2001). Whole body knockout (KO) of p53 results in a decline in mitochondrial protein content, aerobic capacity, and mtDNA (Lebedeva et al., 2009), suggesting that p53 may be an important determinant of mitochondrial content and activity. However, using a muscle‐specific KO, Stocks and colleagues showed that p53 was not essential for mitochondrial density or activity in muscle (Stocks et al., 2017). The dispensable nature of p53 in muscle is further supported by muscle‐specific KO of sirtuin (SIRT) 1, which deacetylates p53. In SIRT1 mKO mice, acetylated p53 increases significantly and yet neither baseline, nor exercise training‐induced increases in, mitochondrial mass and activity were impaired (Philp et al., 2013). This likely is due to the redundancy of signaling pathways that can regulate mitochondrial mass and activity; however, with ageing, it is possible that some of these signals are lost, increasing the necessity for p53.

An increased reliance on fat oxidation also increases adenosine monophosphate (AMP) levels and the phosphorylation of AMP‐activated protein kinase at the threonine 172 (pAMPK^thr172^) site, which in turn regulates fat metabolism through the phosphorylation of ACC and activation of CPT‐1 (Hardie & Pan, 2002) and PGC‐1α (Jäger et al., 2007). Our results show that a KD increases both pAMPK^thr172^ and PGC‐1α in the GTN muscle of female mice (Figure 4b&C) and increases PGC‐1α in the nuclear fraction (Figure 4h). Within the soleus muscle, we observed no difference in levels of PGC‐1α which, is likely attributed to the fact that it is a more oxidative muscle at baseline in middle‐aged mice. Beyond its role in mitochondrial biogenesis and promoting fat oxidation, PGC‐1α is required for the exercise‐induced increase in kynurenine aminotransferase (KAT) proteins in skeletal muscle (Agudelo et al., 2014). KAT proteins are responsible for converting kynurenine (KYN), a byproduct of tryptophan metabolism, to kynurenic acid (KYNA), which cannot cross the blood brain barrier (Fukui et al., 1991). When KYN does enter the brain, it is metabolized into quinolinic acid, a potent neurotoxin (Cervenka et al., 2017). Thus, the conversion of KYN to KYNA is crucial to preventing a variety of inflammatory brain diseases including, Huntington's Disease, Alzheimer's Disease, motor neuron diseases, major psychiatric disorders, and HIV‐associated neurocognitive disorders (Guillemin, 2012). Like exercise, a KD increased KAT protein in skeletal muscle and not the liver. Specifically, KAT1 and KAT4 showed a significant increase and strong trend in the GTN muscle, respectively (Figure 4d,e), whereas KAT4 protein levels alone increased in the soleus muscle (Figure 5d). These results differ somewhat from exercise, since exercise increases expression of all three of the KAT enzymes (1, 3, and 4) in the mouse GTN (Agudelo et al., 2014). Importantly, the increase in KAT levels resulting from exercise training are dependent on PGC‐1α (Agudelo et al., 2014), suggesting that the increase in KAT protein on a KD may result from the greater level of PGC‐1α. The increase in KAT protein on a KD was associated with a significant decrease in KYN and a strong trend to increase KYNA levels in serum (Figure 4j,k), providing a potential mechanism for the improved neurocognitive function on a KD.

Although the effects of KDs on motor function and cognition have been studied in diseased or very young female rodent models (Ruskin et al., 2011, 2017; Van der Auwera et al., 2005), little has been done to evaluate these functions in healthy middle aged female mice fed a KD. Our data show that some measures of locomotor activity, including rearing count and distance covered on the Y maze and elevated plus maze were significantly increased with a KD. This improvement in neurocognitive function was not seen in the middle‐aged male mice on a KD for 2 months (Roberts et al., 2017). Since total movement in the open field, a simple arena consisting of a minimal compartment for the mice to explore, was not altered, the increase in activity in Y and elevated plus maze scores might be driven by a decrease in anxiety and stimulation of exploratory behavior, rather than an elevation in overall movement: another difference between male and female mice fed a KD for 2 months. Muscle motor strength and coordination was not impacted by a short‐term KD in middle aged female mice, and this is consistent with our previous finding in male mice (Roberts et al., 2017). Together, these data suggest that the neurocognitive benefits of a KD may be higher in females.

Although short‐term working memory evaluated using the Y maze or recognition memory measured by novel object test were not altered with the diet, long‐term spatial learning memory assessed with the Barnes maze was improved in middle aged female mice fed a KD for 2 months. This suggests that in female mice a short‐term KD might preferentially impact the hippocampus where long‐term spatial learning memory is centered. Much of the mechanistic focus of the beneficial effects of a KD on cognitive behavior have been hypothesized to occur downstream of improved mitochondrial mass and function within the brain. Interestingly, the improved neurocognitive function observed in female mice on a short‐term KD occurred independent of measurable changes in acetylation or mitochondrial mass within the hippocampus and cortex. This finding could reflect the fact that we did not look at specific regions or cell types within the hippocampus (Levenson et al., 2004) or that the effect on neurocognitive function occurs downstream of a global change in metabolism as a result of the KD. Since improvement in recognition memory was observed in aged male mice on a long‐term KD (Roberts et al., 2017), more studies also need to be done to determine whether a long‐term KD would benefit short‐term working or recognition memory in aged female mice.

## 5 SUMMARY

The data presented here provide crucial insights into the sex specific response to a KD. In contrast to male mice (Roberts et al., 2017), a 2‐month ketogenic diet improves neurocognitive function concomitant with a decrease in circulating kynurenine levels. Like male mice, female mice on a KD increase mitochondrial mass and function in skeletal muscle maybe in response to changes in protein acetylation and PGC‐1α activity. Another potential effect of the increase in PGC‐1α is an increase in kynurenine aminotransferases within muscle and a subsequent decrease in circulating kynurenine and rise in kynurenic acid in the blood. The decrease in circulating kynurenine, and the resulting decrease in the neurotoxin quinolinic acid in the brain, is one potential mechanism for the improved neurocognitive function on a KD in female mice. However, further work is needed to directly test the hypothesis that muscle‐brain crosstalk through the kynurenine aminotransferases and kynurenine contributes to the neurocognitive improvements on a KD.

## AUTHOR CONTRIBUTIONS

J.J.R., K.B., S.P., Z.Z., and J.M.R. contributed to conceptualization of the study. S.J.P., D.S., Z.Z., Y.A, T.T, and J.M.R. contributed to the data collection. S.J.P., Z.Z., D.S., and and K.B. carried out the formal analysis. S.J.P., Z.Z., D.S., K.B, J.M.R., and J.J.R contributed the manuscript. All authors approved the final manuscript.

## CONFLICT OF INTEREST

K. Baar and J Ramsey have received funding to study ketogenic diets from NIH. KB is a Scientific Advisor to KetoKind. Prof Baar has also received grants and donations from other nutritional companies such as PepsiCo, Bergstrom Nutrition, Ynsect, and GelTor.

## Supporting information


Figure S1

## Data Availability

Raw Data were generated at UC Davis. Derived data supporting the findings of this study are available from KB on request.
