# Supplementary figures and images for: 2‐month ketogenic diet preferentially alters skeletal muscle and augments cognitive function in middle aged female mice
Source: Aging Cell. 2022 Sep 23;21(10):e13706. doi: 10.1111/acel.13706 (PMC9577944; doi:10.1111/acel.13706)

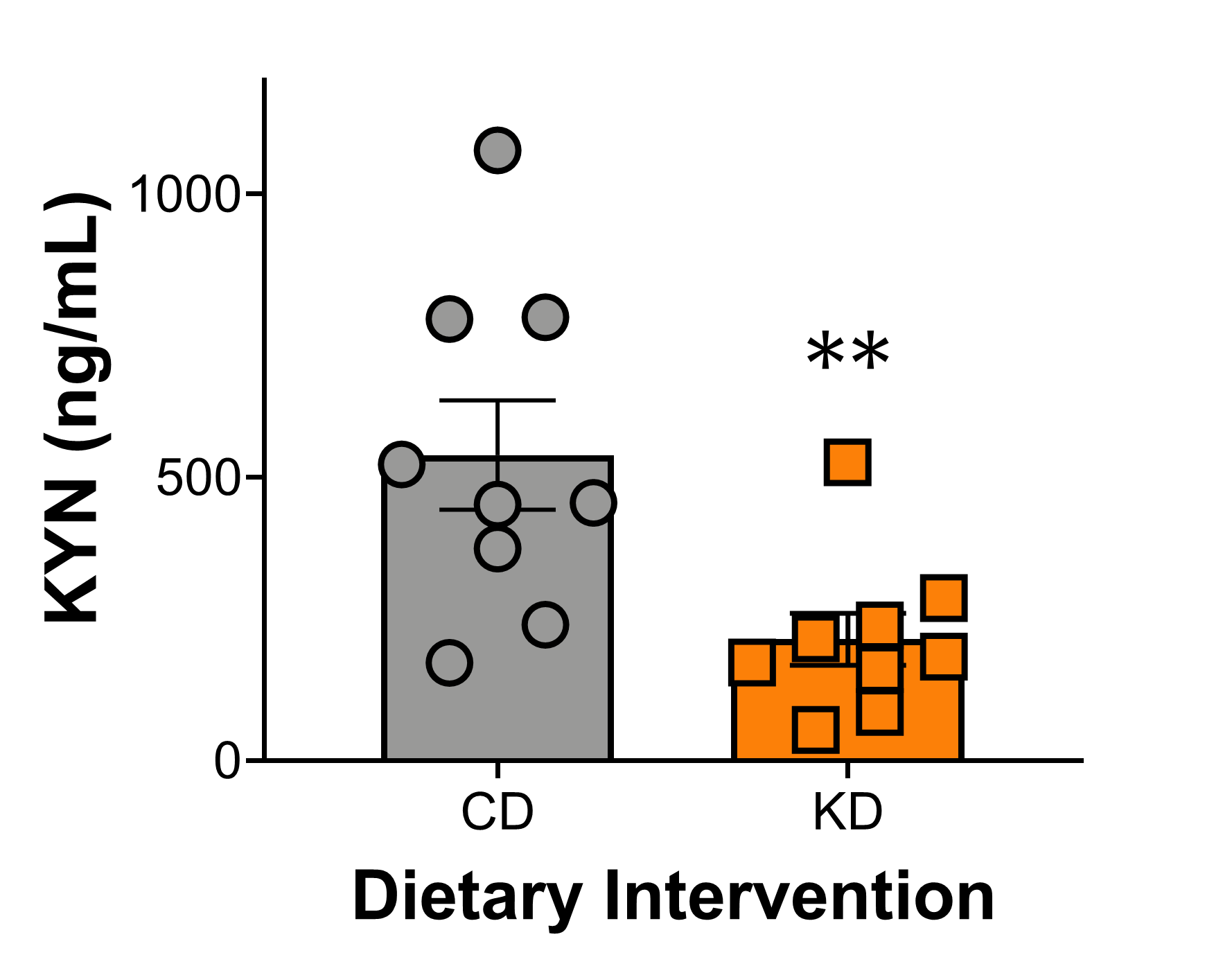

Supplement: Supplementary file 1 — Figure S1 [file ACEL-21-e13706-s001.tif]
